# Effect of plant sterols on the lipid profile of patients with hypercholesterolaemia. Randomised, experimental study

**DOI:** 10.1186/1472-6882-11-73

**Published:** 2011-09-12

**Authors:** Ignacio Párraga, Jesús López-Torres, Fernando Andrés, Beatriz Navarro, José M del Campo, Mercedes García-Reyes, María P Galdón, Ángeles Lloret, Juan C Precioso, Joseba Rabanales

**Affiliations:** 1 Research Unit, Primary Care Head Office of Albacete, Health Care Service of Castilla-La Mancha, Marqués de Villores 6-8, 02001 Albacete, Spain; 2 Almansa Health Centre, Health Care Service of Castilla-La Mancha, C/San Juan s/n, 02640 Almansa, Albacete, Spain; 3 Albacete Area III Health Centre, Health Care Service of Castilla-La Mancha, Plaza La Mancha s/n, 02001 Albacete, Spain; 4 La Roda Health Centre, Health Care Service of Castilla-La Mancha, C/Martínez 63, 02630 La Roda, Albacete, Spain; 5 Pharmacy Service, Primary Care Head Office of Albacete, Health Care Service of Castilla-La Mancha, Marqués de Villores 6-8, 02001 Albacete, Spain

## Abstract

**Background:**

Studies have been conducted on supplementing the daily diet with plant sterol ester-enriched milk derivatives in order to reduce LDL-cholesterol levels and, consequently, cardiovascular risk. However, clinical practice guidelines on hypercholesterolaemia state that there is not sufficient evidence to recommend their use in subjects with hypercholesterolaemia. The main objective of this study is to determine the efficacy of the intake of 2 g of plant sterol esters a day in lowering LDL-cholesterol levels in patients diagnosed with hypercholesterolaemia. The specific objectives are: 1) to quantify the efficacy of the daily intake of plant sterol esters in lowering LDL-cholesterol, total cholesterol and cardiovascular risk in patients with hypercholesterolaemia; 2) to evaluate the occurrence of adverse effects of the daily intake of plant sterol esters; 3) to identify the factors that determine a greater reduction in lipid levels in subjects receiving plant sterol ester supplements.

**Methods/Design:**

Randomised, double-blind, placebo controlled experimental trial carried out at family doctors' surgeries at three health centres in the Health Area of Albacete (Spain). The study subjects will be adults diagnosed with "limit" or "defined" hypercholesterolaemia and who have LDL cholesterol levels of 130 mg/dl or over. A dairy product in the form of liquid yoghurt containing 2 g of plant sterol ester per container will be administered daily after the main meal, for a period of 24 months. The control group will receive a daily unit of yogurt not supplemented with plant sterol esters that has a similar appearance to the enriched yoghurt. The primary variable is the change in lipid profile at 1, 3, 6, 12, 18 and 24 months. The secondary variables are: change in cardiovascular risk, adherence to the dairy product, adverse effects, adherence to dietary recommendations, frequency of food consumption, basic physical examination data, health problems, lipid-lowering medication, physical activity, smoking habits and socio-demographic variables.

**Discussion:**

If plant sterol ester supplements were effective a sounder recommendation for the consumption of plant sterols in subjects with hypercholesterolaemia could be made.

**Trial Registration:**

Current Controlled Trials NCT01406106.

## Background

Cardiovascular disease continues to be the leading cause of death in western countries, and accounts for 33.3% of the total deaths registered in Spain in 2004 [1]. Control of cardiovascular risk factors is imperative for reducing the morbidity and mortality of the population [2]. One of these risk factors is hypercholesterolaemia, which affects 20% of the Spanish population when considering cholesterol levels of ≥ 250 mg/dl, and 50-69% if we consider levels of ≥ 200 mg/dl [3,4]. A linear relationship between relative cardiovascular risk and cholesterol levels in the range of 155 to 310 mg/dL [5,6], and a continuous, gradual relationship between high cholesterol levels and major cardiovascular risk [7,8] have been demonstrated.

The search for a better control of cholesterol levels has instigated numerous studies. Dietary recommendations for reducing cardiovascular diseases have been focussed on the change or reduction in fat or cholesterol intake [9]. One of the strategies for obtaining a tailor-made diet for this disease is the use of foods that contain plant stanols and sterols, which reduce cholesterol absorption [10]. Incorporating plant sterols into the daily diet can lower lipid levels to a similar extent as statins can, in primary prevention [11].

The generic term "functional foods" has been used for all natural or processed food products that provide a benefit beyond smell, flavour, texture or nutrition, and that affect physiological functions in a measurable way in terms of disease prevention or health promotion [12,13]. To definitely confirm the functionality of foods, long term clinical studies must demonstrate that their regular consumption has a clear preventive effect on disease development. There are few controlled studies that demonstrate the beneficial effect of the regular consumption of one complete food [14].

The term phytosterols covers both plant sterols and stanols [15]. They are plant components with a structure similar to cholesterol [16], although they are more poorly absorbed by the intestine. They are classified into different groups depending on their structure and biosynthesis [17]. Their exact mechanism of action and cholesterol lowering properties are not known, but, because their structure is similar to that of cholesterol, they compete for solubilization in the micelles and therefore inhibit intestinal absorption of both dietary and endogenous cholesterol [18]. They are present in vegetable oils as regular food, although they are also available as additives in certain foods such as margarine or yoghurt to obtain a lipid-lowering effect [19].

The regular daily intake of phytosterols ranges from 150-350 mg. A daily dose of at least 1.5 to 3 g/day has been calculated as being the amount needed to achieve a 10-15% reduction in low density lipoprotein cholesterol (LDL-cholesterol). The cholesterol lowering effect appears to level off at higher doses and no significant benefits are obtained [20]. Several studies have demonstrated a reduction in serum total-cholesterol and LDL-cholesterol with the intake of foods containing stanols and sterols [21-23].

The use of sterols combined with statins [24] and fibrates [25] has an added affect on lowering blood cholesterol levels. The favourable effect of triple therapy (statins, sterols and cholestyramine) on cholesterol reduction has also been evaluated [26]. Therefore, sterols can also be useful in the secondary prevention of cardiovascular disease, which requires a greater reduction in LDL-cholesterol.

A meta-analysis of 41 trials with different enriched food products showed that the most recommendable daily intake of phytosterols is 2 g and that this dose reduces LDL-cholesterol by 10% [27]. A dose dependent response relation of up to a dose of 2 g of plant sterol or stanol a day with a reduction in LDL-cholesterol of 15 to 20 mg has also been reported [28]. However, no effect on high density lipoprotein cholesterol (HDL-cholesterol) or triglycerides was found.

Different studies have been conducted on this subject, although very few are long term and with a large number of patients. Mensik et al. [29] performed a controlled trial with 60 patients who consumed low-fat yoghurt enriched with plant stanol esters and observed a decrease in LDL-cholesterol of around 14%, although the dose was higher than 3 g of stanol. Similar results were obtained with margarine in another trial with only 34 patients for 4 weeks [20], in which a daily dose of approximately 2 g reduced LDL-cholesterol by 12% of the initial values. In a longer term study [30], in healthy subjects and patients with familial hypercholesterolaemia treated with statins and sterols, it was observed that the cholesterol-lowering effect was maintained for 2 months.

Other studies have determined that the intake of plant sterols in the range of 1.5 to 2.5 g/day lowers LDL-cholesterol levels by 8.5-10% [27]. However, the efficacy of new formulations to improve the solubility of plant sterols and their effects on lipid reduction, as well as the optimal frequency of their consumption throughout the day, has not yet been investigated. The first studies on the action of phytosterols indicated that in order to optimise their effect they should be taken together with cholesterol-containing food [31]. In the year 2000 a study demonstrated that 2.5 g of plant stanols taken once a day was just as efficacious as the same daily amount divided over 3 meals [32].

The interest in finding new, effective strategies to lower cholesterol and the evidence given above has led several entities to include the consumption of these products in their stepped treatment of hypercholesterolaemia. The dietary recommendations of the USA National Cholesterol Education Programme [33,34] include the consumption of 2-3 g/day of phytostanols. Also the American Heart Association (AHA) [35] recommends foods containing stanol esters for adult patients who need to lower their LDL-cholesterol levels, both in primary and secondary prevention. The AHA considers stanols as a good therapeutic option, in addition to diet and lifestyle changes, for individuals with high LDL-cholesterol levels [36]. This association recommends a daily intake of 2 g of sterols.

Several studies point that there are no significant side effects with the intake of sterols and stanols [28,37,38]. However, it is recommended that the daily phytosterol intake should not exceed 8.6 g/d, as there is a lack of information on the effects of higher intake levels [39]. Phytosterols are therefore considered safe and effective for use as functional food ingredients [40,41]. On the other hand, some studies have described changes in plasma anti-oxidant concentrations [32,42] and a lower absorption of beta carotenes associated to the consumption of phytosterols has been reported [43], although the bioavailability of liposoluble vitamins A, D and E did not appear to be significantly changed. To minimise the possible decrease in plasma carotene levels associated to the consumption of sterols or stanols, the daily diet should include vegetables and fruits [44]. The possible implications for health are considered to be minimal and adverse results are not expected [32]. However, a conservative attitude is recommendable when prescribing for pregnant women, breast-feeding infants and small children. In the European Union, the Scientific Committee on Food has authorised the marketing of phytosterol-enriched margarines, considering their use at levels of up to 8% of free phytosterols, equivalent to 14% of esterified phytosterol, per 100 g of margarine is safe for human use [45].

Although there are some data on the possible atherogenic effect of the phytosterols [46,47], different studies conducted in animal models and humans have demonstrated a reduction in the atheromatous plaque after the administration of plant sterols [48,49]. In animals a decrease in the half life of erythrocytes and an increase in their fragility [50,51] have been observed. The implications of these animal studies for humans are unknown, although De Jong et al. [52] did not observe any changes in the osmotic fragility of the erythrocyte membranes after phytosterol consumption. Furthermore, in the studies included in the meta-analysis by Moruisi et al. [41] only minor digestive side effects were reported that were not confirmed in other studies [53]. In this respect it would also be advantageous to have results of longer term studies.

Most studies on the efficacy of phytosterols have been conducted outside Spain, very probably in populations with life-styles and dietary habits different from those of the Spanish population [37]. This justifies the need to conduct further studies in a Spanish population; especially if we consider that the studies conducted so far included few participants and had a follow-up period of only a few weeks. Rigorous studies are needed that accurately quantify the magnitude of the effects of phytosterols and their interaction with other components of an individual's diet and life-style habits.

Stanols can play an important role in the use of diet as a therapeutic measure. The importance of diet as a therapeutic measure has been recognised for some time (National Cholesterol Education Program Adults Treatment Panel III -NCEP-ATP III-) and has been extensively confirmed by experimental studies in humans [54]. Since the introduction of statins, dietary therapy has received less attention, but the growing concern for the elevated pharmaceutical cost has led to proposals for new alternatives for subjects with high cholesterol levels. It would be useful to determine to what extent combining a lipid-lowering diet with the consumption of sterols would avoid or reduce pharmacological treatment in patients with mild or moderate hypercholesterolaemia.

It has been determined [55] that some persons with high cardiovascular risk could avoid the need for medication to lower their cholesterol levels if they made appropriate changes to their diet. It has been demonstrated that plant sterols and stanols lower such levels, but it is not known if their consumption, as part of a low-fat diet, would provide any clinically significant additional benefit, reducing the frequency of cardiovascular events.

It is appropriate, therefore, to conduct controlled studies to determine the efficacy of plant sterols and stanols in persons with high cholesterol levels (primary prevention). Studies such as the one we propose should determine if plant stanols lower the lipid profile and consequently reduce cardiovascular risk. In accordance with the recommendations of the National Institute for Health and Clinical Excellence of 2008 [55], we will consider a follow up period of 2 years.

### Study objectives

The general objective of our study is to determine the efficacy of the intake of 2 g a day of plant sterol esters in lowering LDL-cholesterol in patients diagnosed with hypercholesterolaemia.

The specific objectives are: 1) to quantify the effect of the daily intake of plant sterol esters in lowering LDL-cholesterol in patients with hypercholesterolaemia at 1, 3, 6, 12, 18 and 24 months; 2) to quantify the effect of the daily intake of plant sterol esters in lowering total cholesterol in patients with hypercholesterolaemia at 1, 3, 6, 12, 18 and 24 months; 3) to quantify the effect of the daily intake of plant sterol esters in reducing cardiovascular risk in patients with hypercholesterolaemia at 1, 3, 6, 12, 18 and 24 months; 4) to evaluate the occurrence of adverse effects of the daily intake of plant sterol esters; 5) to identify the factors that determine a greater reduction in lipid levels in subjects receiving plant sterol ester supplements, related to the patient's health, dietary habits and socio-demographic variables.

## Methods/Design

### Design

Randomised, double-blind, placebo-controlled experimental trial to determine the efficacy of plant sterol esters as dietary supplement in reducing lipids.

### Study setting and subjects

Patients diagnosed with hypercholesterolaemia will be recruited from 9 family doctors' surgeries at 3 healthcare centres in the health care area of Albacete, Spain.

The inclusion criteria are: a) subjects diagnosed with limit hypercholesterolaemia (total cholesterol 200-249 mg/dl) or defined hypercholesterolaemia (total cholesterol equal to or above 250 mg/dl) who have LDL-cholesterol levels equal to or above 130 mg/dl, b) subjects aged 18 years or over attending the participating health centres, c) subjects who give their consent to participate after being informed of the study objectives. The exclusion criteria are: a) known hypersensitivity to sterol esters or to the other components of the food that contains them (liquid yoghurt), b) contraindication for treatment with sterol esters or any of the components of the food, c) physical disability that hinders collaboration, d) significant chronic organic or psychiatric disease, e) not obtaining informed consent.

### Sample size

To obtain a 90% statistical power with an alpha error of 0.05 (bilateral hypothesis). The effect to be demonstrated is based on the following:

- The expected mean value of LDL-cholesterol in the patients included in the study is 157 mg/dl ± 30 SD (value obtained in previous studies conducted in a Spanish population).

- To demonstrate an effect of a 10% reduction in LDL-cholesterol in patients who receive the supplements, 152 subjects are needed (76 in the active group and 76 in the placebo group).

Assuming a 20% loss to follow up, 182 subjects will need to be selected and will be distributed equally between the two groups in order to obtain the maximum statistical power.

The recruitment period will be from January 2010 to September 2010.

### Formation of groups and blinding

The subjects will be randomly and equiprobably assigned to the experimental or the control group. The randomization will be generated by computer, with random numbers following a size 4 block system (ensures heterogeneity of the diet supplement administered in the consecutive patient groups, ensuring that in each interval exists a similar number of patients in each group). Randomisation will be done centrally.

The containers of yoghurt (with or without the plant sterol ester supplement) will be given to the patients in a blinded manner. To protect the blinding, the container of the placebo and test product will be exactly the same.

To ensure objectivity in the interpretation of the results, the study will be conducted in a blinded manner for the patients, investigators and data analysts. The placebo and the active product will be identical in appearance and will be identified by a code, the assignment of which will be unknown by the patients and the investigators.

### Intervention

The administration of a dairy product in the form of liquid yoghurt, marketed in Spain, that contains 2 g per container of plant stanol esters: sitostanol and campestanol (AHA recommended dose - 1.5 to 3 g). The enriched product and the placebo will have the same characteristics (composition and outward appearance), but the placebo will not contain stanol esters. The dose will be one container a day, after the main meal, for 24 months. The participants may continue with their previously prescribed lipid-lowering treatment and new treatment needed for this disease or for other diseases. Composition per container: proteins 1.8 g, carbohydrates 9.8 g, fat (except stanol) 1.4 g, plant stanol 2 g, vitamin B6 0.6 mg, folic acid 60 mg. The control group will receive one unit a day of yoghurt not supplemented with stanol esters that has a similar appearance to the enriched yoghurt.

### Follow-up

The subjects will be followed-up for two years.

- Selection: patients who meet the inclusion criteria will be asked to participate in the study. After giving their informed consent they will be given an appointment for the initial visit and for the analytical tests.

- Initial visit: the participant will be randomised to one of the two groups. Their medical history will be taken and they will be given a physical examination. The corresponding dairy product will be dispensed (the following doses will be dispensed according to their expiry date). At this visit a lipid-lowering diet will be recommended to all the participants.

- Follow-up visits: at 1, 3, 6, 12, 18, and 24 months, the physical examination and analytical test parameters will be recorded. The cardiovascular risk will be calculated and possible adverse effects and compliance or adherence to the food supplied will be recorded.

The study will be finalised for the patient in the following circumstances: completed follow-up period (2 years), death, violation of the protocol, adverse event, intercurrent disease that makes it impossible to ingest food and patient abandons study or withdraws consent.

### Primary variables and measuring methods

#### Primary variable

-Change in lipid profile at 1, 3, 6, 12, 18 and 24 months. Lipid levels will be measured in both plasma and capillary blood at the initial visit. The validity of the capillary blood lipid levels can be checked with the Cardiochek analyser (by total cholesterol, HDL-cholesterol and triglyceride strips, and subsequent calculation of LDL-cholesterol using the Friedewald formula). The measurements at 3, 12 and 24 months will be in plasma. At 1, 6 and 18 months they will only be in capillary blood.

#### Secondary variables

-Change in cardiovascular risk at the follow-up visits. Systematic Coronary Risk Evaluation (SCORE) and Gerona Heart Register (REGICOR) tables will be used for the evaluation.

-Adherence to the dairy product (liquid yoghurt) by self-report and Morisky-Green scale, which determines the degree of coincidence between the patient's behaviour and the doctor's advice (a non-complier is one who answers one of the four questions of the scale inappropriately).

-Adverse events. Considered as any undesirable event in any patient included in the study, even though it does not have a causal relation with the product. Known adverse events of phytosterols in the diet at the proposed doses: occasionally mild digestive alterations.

-Adherence to the dietary recommendations: 5-point Likert scale.

-Frequency of food intake using the CDC-FFQ questionnaire, validated in Spain (Aguirre, 2008) considered appropriate to classify the subjects according to their intake of food and nutrients.

-Occurrence of cardiovascular events during the follow-up period: ischaemic heart disease, atherothrombotic cerebrovascular disease, heart failure and peripheral artery disease.

-Weight, height, body mass index (BMI): classification of subjects according to degree of obesity.

-Physical activity: amount of aerobic exercise ("active" if performs aerobic exercise for 30 minutes or more, three or more times a week, "partially active" if exercises with less frequency and for less time that this and "inactive" when does not perform any type of exercise).

-Smoking habit: considered smoker if answers yes to the question "do you smoke?"

-Systolic and diastolic blood pressure (two measurements): the result will be the mean of the two measurements.

-Health problems (WONCA ICPC-2).

-Whether taking lipid-lowering pharmacological treatment.

-Socio-demographic data: age, gender, marital status, educational level and social class based on occupation (National Classification of Occupations).

### Statistical analysis

#### Test of the hypothesis

The null hypothesis establishes that there is no relationship between the decrease in blood LDL-cholesterol levels and the consumption of stanol ester enriched liquid yoghurt. The alternative hypothesis establishes a relationship between such consumption and the incidence of the primary variable.

#### Efficacy analysis

After the prior stages of data purification, exploratory analysis and categorisation or transformation of variables, the following were performed:

-Comparison of the variables of interest and the stratification and potentially confounding variables in both groups at the start of the study. Although we used a random assignment system, we checked that the baseline values of the study variables were uniform over the two groups.

-Classification of the subjects of the two groups into different LDL-cholesterol and total cholesterol reduction levels. A baseline raw analysis will be performed to evaluate the following parameters and their 95% confidence intervals: ABI (absolute benefit increase), RBI (relative benefit increase) and NNT (number needed to treat). A stratified analysis will be performed adjusted for each independent variable.

-The incidence of the endpoint variables in the two groups will be described and compared at each follow-up period (comparison of means using Student's t tests or Mann-Witney U test, depending on the distribution of the variables). The Friedman test will be used to compare the changes in lipid profile throughout the study in each of the groups.

-The effect of the dairy product on total cholesterol and LDL-cholesterol levels will be determined, adjusted for changes in adherence to the prescribed regimen (linear models generalised for repeated means).

-The convergent validity of the capillary blood measurements compared to the plasma measurements will be analysed through intraclass correlation.

**Figure 1:**
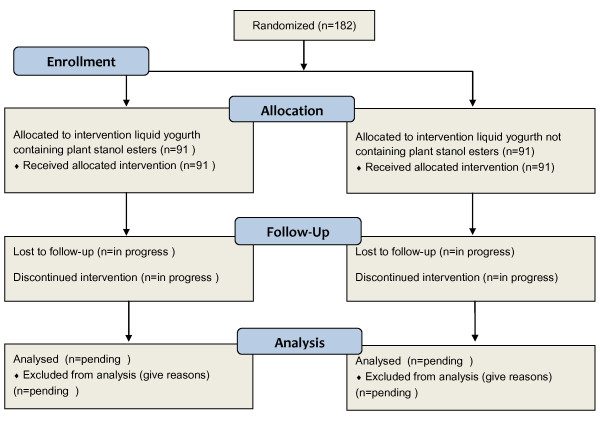
**Study's flowchart outlining**.

The analysis will be an intention to treat analysis including all randomised patients in the efficacy analysis, according to their group and regardless of the treatment received. A per-protocol analysis will also be performed for the primary variables. Intermediate analyses will be performed to decide if the study should be prematurely terminated in the event of frequent adverse effects or a clear improvement in the test group (see Figure 1 for the study flow chart).

### Ethics Approval

This project was approved by the Research Ethics Committee of the Universitary Hospital of Albacete on May 27, 2009

## Discussion

The results of this study will provide valuable information on the efficacy of plant sterol ester supplements in preventing cardiovascular disease by lowering plasma cholesterol levels and consequently cardiovascular risk. If this supplement were effective a sounder recommendation for the consumption of plant sterols in subjects with hypercholesterolaemia could be made.

Possible limitations of the study - using a randomisation procedure reduces the risk of selection bias as the investigator will not assign treatment and any factor that effects participation will be balanced in the two groups. Confounding will also be reduced as randomisation tends to divide the determinant factors, which influence the final result, equally between the two groups. However, bias could exist on collecting information due to the regression towards the mean effect or tendency of the continuous variables towards the mean in successive measurements, applicable to the analytical results. There is also a possible risk of differential loss to follow up between the two groups, due to the length of the study and also to the placebo effect which could contribute to the patients abandoning the study because they consider that the treatment provided is not having any effect.

The study protocol has been evaluated and approved by the Institutional Review Board of the Albacete Health Area on 23rd April, 2009. During the performance of the study the following ethical principles will be respected: voluntary participation with consent of the patient, guarantee of anonymity in the information provided by the patient, data provided by the patient exclusively restricted to use in the proposed study. The investigators will ensure that the study is conducted in accordance with the declaration of Helsinki and with legislation in effect (Royal Decree 223/2004) and the New Code of Medical Ethics of the Spanish Medical Association. The study will fully comply with Good Clinical Practice principles.

Finally, for the development of this project the following principles have been considered: 1) data on the dairy product are sufficient to guarantee that the risk to the study participants is acceptable; 2) the objectives of the study will probably provide further knowledge of the topic to be studied; 3) the available information warrants the performance of this study and its potential risk to the participants.

## Conflicting interests

The authors declare that they have no competing interests.

## Authors' contributions

IP and JL-T are the principal investigators responsible for the development of the project and writing the protocol. JL-T was responsible for the study design, estimation of the sample size and the statistical analyses. FA, BN JC, MG-R, PG, ALl, JP and JR have contributed to the description of the background, the general design and the definition of the different study variables and their adaptation to the various computerised clinical recording systems.

All the authors have read and approved the final version of the protocol.

## Pre-publication history

The pre-publication history for this paper can be accessed here:

http://www.biomedcentral.com/1472-6882/11/73/prepub
